# NKp30 expression is a prognostic immune biomarker for stratification of patients with intermediate-risk acute myeloid leukemia

**DOI:** 10.18632/oncotarget.17747

**Published:** 2017-05-10

**Authors:** Anne-Sophie Chretien, Cyril Fauriat, Florence Orlanducci, Jerome Rey, Gaelle Bouvier Borg, Emmanuel Gautherot, Samuel Granjeaud, Clemence Demerle, Jean-François Hamel, Adelheid Cerwenka, Elke Pogge von Strandmann, Norbert Ifrah, Catherine Lacombe, Pascale Cornillet-Lefebvre, Jacques Delaunay, Antoine Toubert, Christine Arnoulet, Norbert Vey, Daniel Olive

**Affiliations:** ^1^ Team Immunity and Cancer, Centre de Recherche en Cancérologie de Marseille (CRCM), Inserm, U1068, CNRS, UMR7258, Institut Paoli-Calmettes, Aix-Marseille University, UM 105, Marseille, France; ^2^ Immunomonitoring Platform, Institut Paoli-Calmettes, Marseille, France; ^3^ Hematology Department, Centre de Recherche en Cancérologie de Marseille (CRCM), Inserm, U1068, CNRS, UMR7258, Institut Paoli-Calmettes, Aix-Marseille University, UM 105, Marseille, France; ^4^ Beckman Coulter Immunotech, Marseille, France; ^5^ Systems Biology Platform, Centre de Recherche en Cancérologie de Marseille (CRCM), Inserm, U1068, CNRS, UMR7258, Institut Paoli-Calmettes, Aix-Marseille University, UM 105, Marseille, France; ^6^ Biostatistics and Methodology Department, CHU Angers, Angers, France; ^7^ Innate Immunity Group, German Cancer Research Center, Heidelberg, Germany; ^8^ Department I of Internal Medicine, University Hospital of Cologne, Cologne, Germany; ^9^ Hematology Department, CHU Angers, Angers, France; ^10^ GOELAMStheque, FILO French Innovative Leukemia Organization, Cochin Hospital, APHP, Paris, France; ^11^ Laboratoire d'Hématologie, Centre Hospitalier Universitaire de Reims, Reims, France; ^12^ Service d'Hématologie, Centre Catherine de Sienne, Nantes, France; ^13^ INSERM UMRS-1160, Univ Paris Diderot, Sorbonne Paris Cité, Institut Universitaire d'Hématologie, Immunology and Histocompatibility Department, Hôpital Saint-Louis, APHP, Paris, France; ^14^ Biopathology Department, Institut Paoli Calmettes, Marseille, France; ^15^ Clinic for Hematology, Oncology and Immunology, Experimental Tumor Research, Center for Tumor Biology and Immunology, Philipps University, Marburg, Germany

**Keywords:** AML, prognostic biomarkers, natural killer, NCR, NKp30

## Abstract

Cytogenetics and European Leukemia Net (ELN) genetic classification predict patients at increased risk of relapse in acute myeloid leukemia (AML) except in the intermediate risk group for which further prognostic determinants are required. We have previously shown that Natural Killer (NK) cell defects in AML are predictors of poor overall survival (OS). This study aimins at validating NKp30, a receptor that mediates NK activation, as a prognostic biomarker for AML patients with intermediate prognosis.

NKp30 expression was prospectively assessed at diagnosis on NK cells from peripheral blood by flow cytometry (*N* = 201 patients). Clinical outcome was evaluated with regard to NKp30 status.

In patients with intermediate cytogenetic (*N* = 162), NKp30^high^ phenotype at diagnosis was predictive of better OS (HR = 0.26; 95%CI = [0.14-0.50]; *P* < 0.0001) and relapse-free survival (RFS) (HR = 0.21; 95%CI = [0.08-0.52]; *P* = 0.0007). In patients with intermediate ELN (*N* = 116), NKp30^high^ phenotype at diagnosis was predictive of better OS (HR = 0.33; 95%CI = [0.16–0.67]; P = 0.0019) and RFS (HR = 0.24; 95%CI = [0.08-0.67]; *P* = 0.0058). In multivariate analysis, high NKp30 expression independently predicted improved OS (HR = 0.56, *P* = 0.046) and RFS (HR = 0.37, *P* = 0.048). Consistently, cumulative incidence of relapse (CIR) was lower in patients with high NKp30 expression (HR = 0.37, *P* = 0.026).

In conclusion, we propose NKp30 status as a simple and early prognostic biomarker that identifies intermediate-risk patients with poor prognosis who otherwise may not be identified with existing risk stratification systems.

## INTRODUCTION

Patient stratification at diagnosis for acute myeloid leukemia (AML) is crucial for clinical decision making regarding post-remission therapy. To date, patient stratification is based on cytogenetics and molecular classifications [1, 2]. As proposed by the ELN genetic classification based on FLT3/CEBPα/NPM1 mutational status further refines patient stratification, but clinical uncertainty remains with an unclassifiable group of patients with intermediate prognosis [3, 4].

New molecular markers have been shown to impact prognosis and have been included in the revision of ELN classification [3, 5–7]. However, molecular markers do not account for the entire prognostic heterogeneity of AML and new markers are warranted. In this context, accurate estimation of the risk of relapse at diagnosis or after complete remission (CR) in patients with intermediate prognosis is essential for physicians in order to evaluate the potential benefits of intensive chemotherapy and allogeneic stem cell transplantation (allo-SCT).

Among candidate biomarkers, immune parameters are currently extensively evaluated in immunomonitoring studies. Among immune effectors implicated in immune surveillance in AML, Natural Killer (NK) cells are of particular importance. NK cells are key components of the innate immunity and substantially contribute to the anti-tumor immune responses, in particular in the context of AML [8, 9]. NK cells are crucial immune effectors and play a key role in tumor rejection, with direct effect on tumor cells as well as an important role in regulation of the adaptive immune response through the cross-talk with antigen presenting cells [10]. NK cells prevent emergence of transformed cells, and are involved in response to chemotherapy and radiotherapy [11–13]. In AML, their direct effect on the tumor burden is illustrated by the success of hematopoietic stem cell transplantation with KIR-HLA mismatch in hematologic malignancies [14–17]. NK cell anti-tumor activity is triggered by NK activating receptors, including Natural Cytotoxic Receptors (NCR) such as NKp30 [18, 19]. Decay in anti-tumor activity of NK cells, related to defective activating NK receptor expression has been described in several cancers [18, 20–22]. In particular, it has been previously shown that NCR expression at diagnosis is a potential discriminant biomarker in AML and in solid tumors [18, 20, 22]. Hence, our group previously reported that low NKp30 expression on NK cells was significantly associated with reduced survival in AML [18]. Therefore, NKp30 represents a potential biomarker for refinement of patient stratification, and might identify AML patients with high risk of relapse among patients with intermediate prognosis, with potential improvement of therapeutic decision algorithms. The present study was designed to address this question.

## RESULTS

### Baseline patient characteristics

The patient characteristics, stratified by NKp30 expression groups, are summarized in Table 1. 21 patients had favorable cytogenetics (10.4%), 162 had intermediate cytogenetics (80.6%) and 18 had unfavorable cytogenetics (9.0%). The mean age (± SD) at induction was 46.7 years (± 10.8). Median follow-up after diagnosis was 26.31 (± 1.6) months. Cytogenetic classification and ELN genetic classification [3] (FLT3/CEBPα/NPM1 mutational status) were routinely determined in the Biopathology departments of the centers involved in this study.

**Table 1:** Baseline patients characteristics (1/2)

Characteristic		All	NKp30 low	NKp30 high
**Patients, no**.	*N* (%)	201 (100)	55 (27.4)	146 (72.6)
**Age at diagnosis**	Mean (SD)	46.7 (10.8)	47.7 (11.4)	46.4 (10.6)
**Sex ratio, M/F**		1.09	1.42	0.91
**FAB category**	*N* (%)			
**M0**		2 (1.0)	0 (0.0)	2 (1.4)
**M1**		28 (13.9)	8 (14.5)	20 (13.7)
**M1/2**		2 (1.0)	1 (1.8)	1 (0.7)
**M2**		39 (19.4)	8 (14.5)	31 (21.2)
**M3**		0 (0.0)	0 (0.0)	0 (0.0)
**M4**		56 (27.9)	16 (29.1)	40 (27.4)
**M4/5**		1 (0.5)	0 (0.0)	1 (0.7)
**M5**		49 (24.4)	16 (29.1)	33 (22.6)
**M6**		9 (4.5)	1 (1.8)	8 (5.5)
**M7**		0 (0.0)	0 (0.0)	0 (0.0)
**Unclassified**		5 (2.5)	3 (5.5)	2 (1.4)
**NA**		10 (5.0)	2 (3.6)	8 (5.5)
**Status at diagnosis**	*N* (%)			
**de novo**		181 (90.0)	46 (83.6)	135 (92.5)
**s-AML**				
**MDS**		17 (8.5)	8 (14.5)	9 (6.2)
**t-AML**		3 (1.5)	1 (1.8)	2 (1.4)
**White blood cell (10^9^ cells/L)**	Median (SD)	18.8 (62.3)	30.0 (82.5)	13.9 (44.8)
**Cytogenetic prognosis**	*N* (%)			
**Favorable**		21 (10.4)	9 (16.4)	12 (8.2)
**Intermediate**		162 (80.6)	42 (76.4)	120 (82.2)
**Adverse**		18 (9.0)	4 (7.3)	14 (9.6)
**Mutations in intermediate cytogenetics group**	*N* (%)			
**Done**		158	39	119
**FLT3 ITD mut**		53/158 (33.5)	12/39 (30.8)	41/119 (34.5)
**NPM1mut**		75/158 (47.5)	15/39 (38.5)	60/119 (50.4)
**CEBPαmut / FLT3wt NPM1wt**		11/64 (17.2)	2/19 (10.5)	9/45 (20.0)
**ELN**	*N* (%)			
**Favorable**		67 (33.3)	18 (32.7)	49 (33.6)
**Intermediate**		116 (57.7)	33 (60.0)	83 (56.8)
**Intermediate-I**		71 (9.0)	15 (27.3)	56 (38.4)
**Intermediate-II**		41 (20.4)	17 (30.9)	24 (16.4)
**NA**		4 (2.0)	1 (1.8)	3 (2.1)
**Adverse**		18 (9.0)	4 (7.3)	14 (9.6)
**Blasts (blood) at diagnosis**	Mean (SD)	47.3 (31.9)	52.8 (33.1)	45.1 (31.3)
**Blasts (BM) at diagnosis**	Mean (SD)	64.5 (23.2)	70.2 (20.0)	62.3 (24.0)
Response at d15	*N* (%)	116 (57.7)	25 (45.5)	91 (62.3)
No response at d15		57 (28.4)	17 (30.9)	40 (27.4)
NA (Not evaluable or induction death)		28 (13.9)	13 (23.6)	15 (10.3)
Post induction CR	*N* (%)	169 (84.1)	42 (76.4)	127 (87.0)
No post induction CR		32 (15.9)	13 (23.6)	19 (12.3)
Induction death		13 (6.5)	7 (12.7)	6 (4.1)
No CR achieved		19 (9.5)	6 (10.9)	13 (8.9)
Nb of induction for CR	*N* (%)			
1		124 (61.7)	29 (52.7)	95 (65.1)
2		39 (19.4)	11 (20.0)	28 (19.2)
3		6 (3.0)	2 (3.6)	4 (2.7)
Consolidation	*N* (%)			
Chemotherapy +/−Auto-SCT		118 (58.7)	28 (51.0)	90 (61.6)
Chemotherapy +Allo-SCT		83 (41.3)	27 (49.1)	56 (38.4)
Median OS (months)		40.38	21.62	55.98
Median RFS (months)		41.86	10.61	> 60

Allo-SCT: allogeneic stem cell transplantation; Auto-SCT: autologous stem cell transplantation; BM: bone marrow; CR : complete remission; FAB: French-American-British classification; M : male; F : female; ITD: internal tandem duplication; MDS: myelodysplastic syndrome; NA : not available; Nb: number; t-AML: therapy-related AML; s-AML; secondary AML; OS: overall survival; RFS: relapse-free survival.

Baseline NKp30 expression on Natural Killer (NK) cells was assessed by flow cytometry. Among the 201 patients, 146 (72.6%) had NKp30^high^ phenotype, and 55 (27.4%) had low NKp30^low^ phenotype (Table 1). The frequency of patients with NKp30^high^ and NKp30^low^ phenotype did not differ between age, cytogenetics number of inductions, sex or post remission therapy (chemotherapy ± allo-SCT).

### NKp30 status stratifies patients with intermediate prognosis

**Figure 1:**
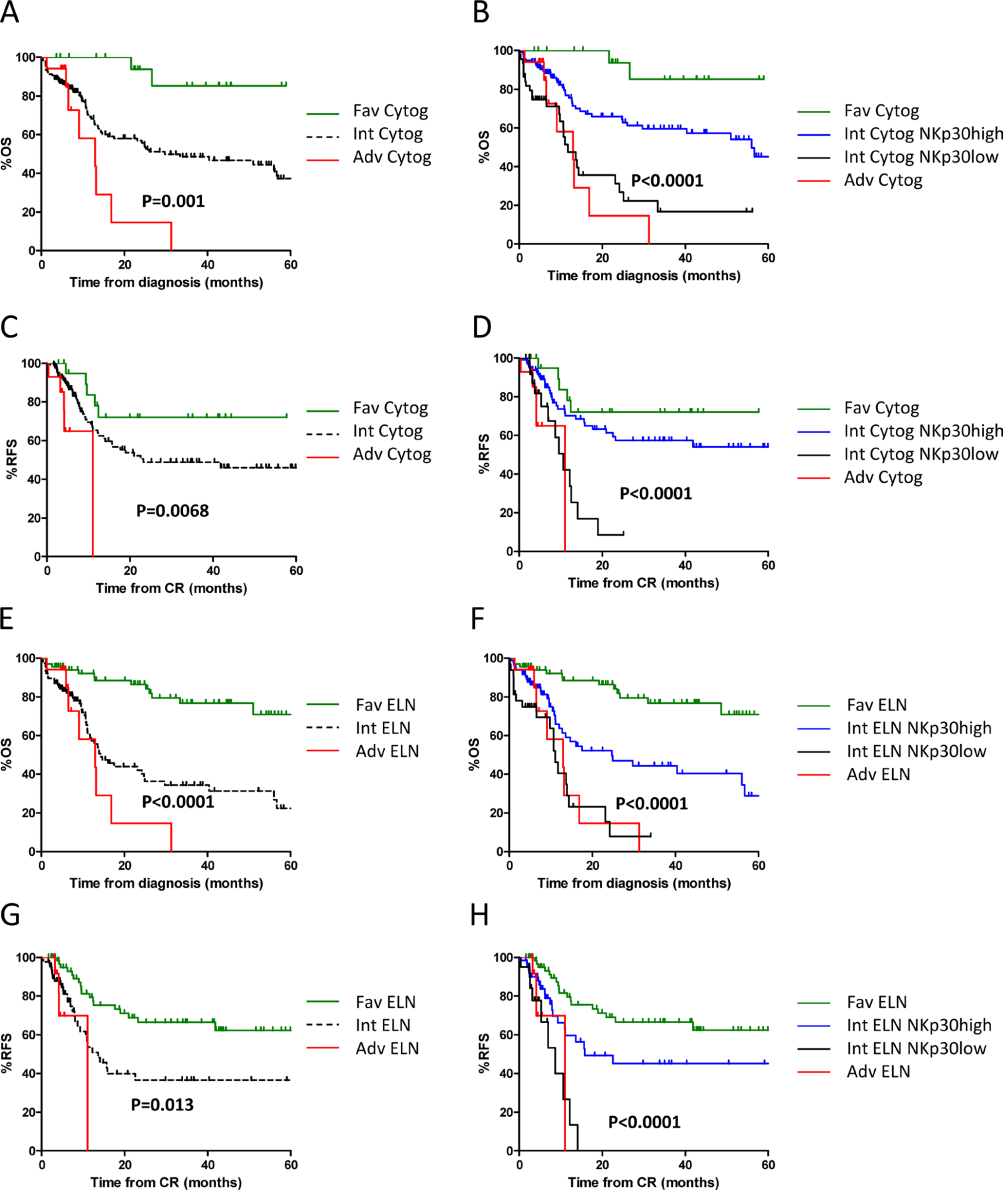
NKp30 stratifies patients with intermediate prognosis Patients were stratified by cytogenetic prognosis. (**A** and **C**) and ELN (**E** and **G**). Patients with intermediate prognosis were re-classified according to NKp30 status (**B**, **D**, **F** and **H**). Panels A, B, E, F display Kaplan-Meier estimates of OS. Panels C, D, G, H display Kaplan-Meier estimates of RFS. Statistical analyses were performed using a log Rank tests. *P* < 0.05 was considered significant. Adv: adverse; Fav: favorable; ELN: European Leukemia Network classification; Int: intermediate; OS: overall survival; RFS: relapse-free survival.

An important challenge is to stratify AML patients with intermediate cytogenetic prognosis. In the group of patients with intermediate cytogenetic prognosis, patients with NKp30^high^ phenotype at diagnosis had better OS (HR = 0.26; 95%CI = [0.14–0.50]; *P* < 0.0001) (Figure 1B) and RFS (HR = 0.21; 95%CI = [0.08–0.52]; *P* = 0.0007) (Figure 1D) than patients with NKp30^low^ phenotype, with better 3-year OS and RFS rates for the group with NKp30^high^phenotype (59.5% vs 16.7% and 57.3% vs 8.5%, respectively).

Insofar as stratification at diagnosis is currently based on cytogenetic classification and completed with ELN genetic classification, we hypothesized that NKp30 status could refine this stratification, particularly in the group of intermediate prognosis. We assessed the prognostic value of NKp30 in patients with intermediate ELN. In these patients, NKp30 significantly risk-stratified patients, with lower OS (HR = 0.33; 95%CI = [0.16–0.67]; *P* = 0.0019) and RFS (HR = 0.24; 95%CI = [0.08–0.67]; *P* = 0.0058) in patients with low NKp30 expression (Figure 1F and 1H, respectively), and better 3-year OS and RFS rates for the group with NKp30^high^ phenotype (44.3% vs 7.7% and 45.7% vs 0%, respectively).

**Figure 2:**
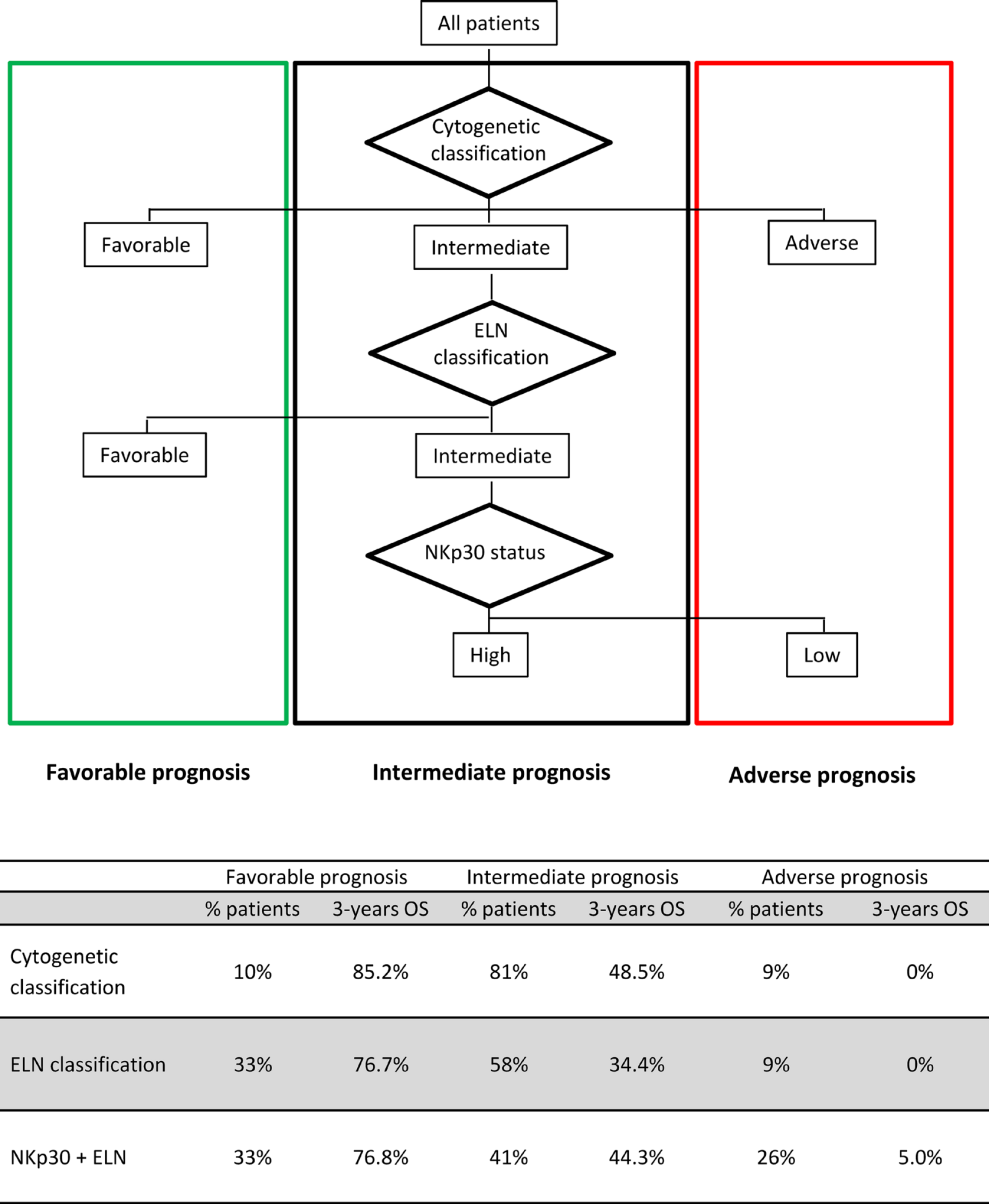
Proposed risk stratification algorithm based on cytogenetic and ELN risk classification refined by NKp30 status When incorporating NKp30 expression in the integrated risk classification of AML based on cytogenetic classification and mutational status (ELN classification), 17% patients (29% of intermediate ELN patients) were re-classified in the unfavorable-risk group.

Thus, NKp30 better discriminates patients in the intermediate group, with survival probability comparable to survival of patients with adverse prognosis based on the reference classifications (Figures 1 and 2) and sub-stratifies one third of patients with intermediate ELN (Figure 2).

In the Cox regression model, high NKp30 expression was significantly associated with improved OS and RFS in patients with intermediate ELN, independent of other factors (HR = 0.56, *P* = 0.046 and HR = 0.48, *P* = 0.048, respectively), even when accounting for the potential effect of allo-SCT (Table 2). Consistently, CIR was lower in patients with high NKp30 expression (HR = 0.37; *P* = 0.026) (Table 2).

**Table 2:** Cox regression

Variable	Multivariate HR for OS			Multivariate HR for RFS			Multivariate HR for CIR		
	HR	95% CI	*P*	HR	95% CI	*P*	HR	95% CI	*P*
**Age at diagnosis**									
≥ 50	Reference			Reference			Reference		
< 50	1.16	.68 to 1.97	.578	1.25	.66 to 2.35	.498	1.12	.51 to 2.42	.773
**Disease status**									
MDS/t-AML	Reference			Reference			Reference		
De novo	.39	.17 to .87	.021	.78	.27 to 2.24	.643	.98	.25 to 3.80	.979
Leucocytosis at diagnosis									
<50 G/L	Reference			Reference			Reference		
≥50 G/L	.88	.51 to 1.52	.655	.92	.48 to 1.75	.800	.67	.31 to 1.42	.297
**Consolidation**									
No allo-SCT	Reference			Reference			Reference		
Allo-SCT	.44	.23 to .84	.013	.48	.23 to .98	.042	.210	.08 to .51	.0006
**NKp30 MFI ratio**									
Low	Reference			Reference			Reference		
High	.56	.31 to .99	.046	.48	.23 to .99	.048	.37	.15 to .89	.026

Abbreviations: allo-SCT: allogeneic stem cell transplantation in 1^st^ complete remission; CI: confidence interval; CIR: cumulative incidence of relapse; RFS: relapse-free survival; ELN: European Leukemia Net genetic classification; HR: hazard ratio; MDS: myelodysplastic syndrome; OS: overall survival; t-AML: therapy-related AML.

Multivariate Cox regression models were used to assess the predictive value of NKp30 expression in patients with intermediate ELN while adjusting for the prognostic factors in the population (age at diagnosis, disease status, leukocytosis, and allogeneic stem cell transplantation as a time-dependent covariate).

Model discrimination improvement was assessed by the c-index (IPC cohort). The model based on NKp30 combined with ELN had higher probability of correctly predicting survival (c-index = 0.716) compared with ELN alone (c-index = 0.683) or cytogenetic classification (c-index = 0.598).

### NKp30 recovery after CR is a better predictor of clinical outcome than NKp30 at diagnosis

**Figure 3:**
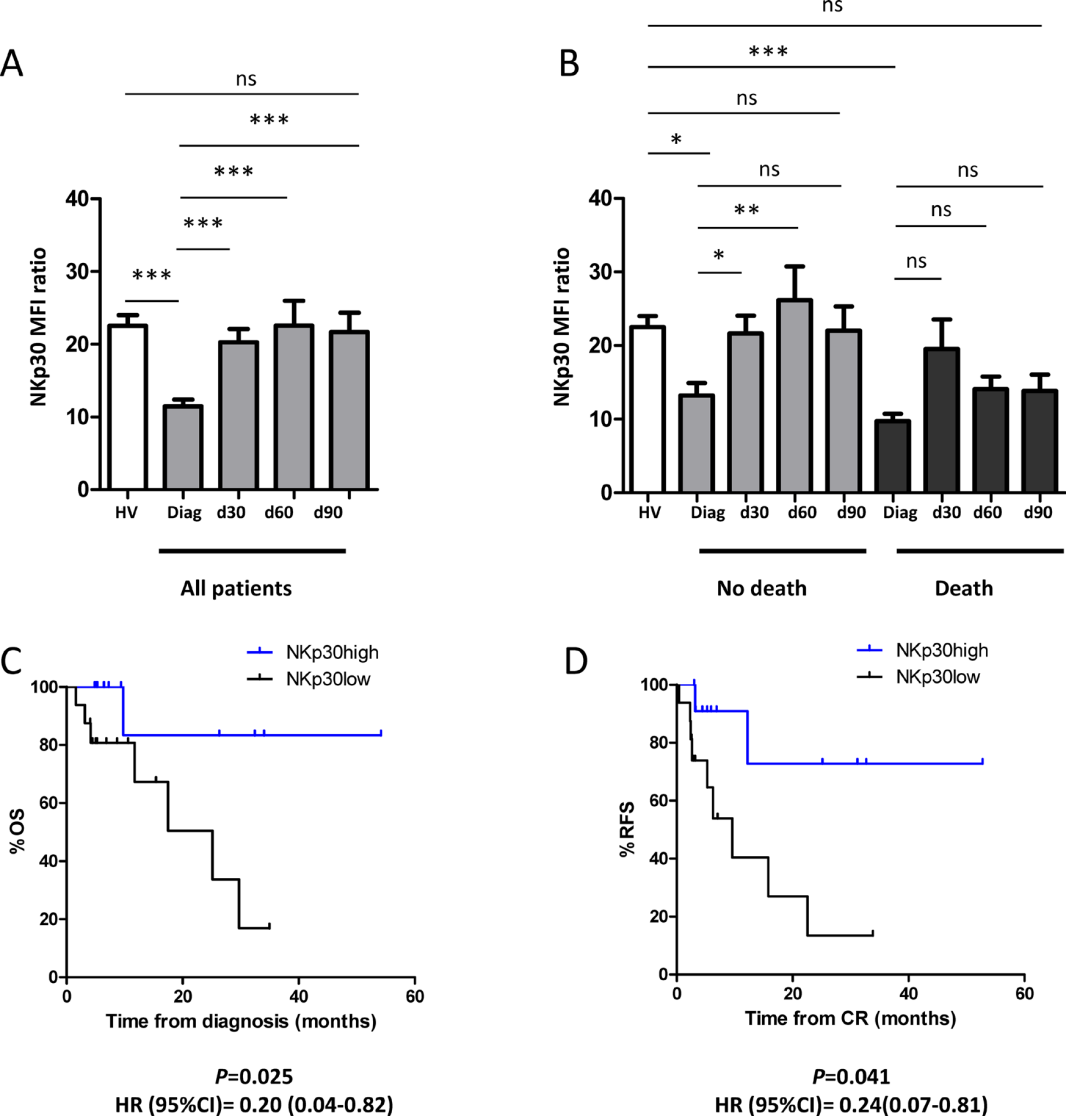
NKp30 recovery after CR is a better predictor of clinical outcome than NKp30 at diagnosis (**A**) The kinetics of NKp30 expression was assessed after CR (day 30, 60 and 90 after the last induction chemotherapy) in the IPC prospective cohort. Thirty-nine patients were tested for NKp30 expression at day 30, 28 patients at day 60 and 23 patients at day 90. The results were compared with NKp30 expression in healthy volunteers (*N* = 34) (**B**) Kinetics of NKp30 expression according to clinical outcome at 2 years. Kaplan-Meier estimates for overall survival (**C**) and relapse-free survival (**D**) by NKp30 expression at day 30 after induction chemotherapy in patients with intermediate cytogenetic prognosis (*N* = 28). Statistical analyses were performed using a Kruskal-Wallis test was used followed by a Dunn's pos*t*-test, and a log Rank tests for survival analyses. *P* < 0.05 was considered significant. 95%CI: 95% confidence interval; Diag: diagnosis; CR: complete remission; HR, hazard ratio; HV: healthy volunteers; ns: non significant.

NKp30 expression was assessed in 39 patients from the IPC prospective cohort in CR after chemotherapy, regardless of ELN group. Induction chemotherapy resulted in significant increase of NKp30 expression at day 30 that was maintained at least until day 90 (Figure 3A). We then divided patients in two groups based on presence or absence of death 2 years after diagnosis. In the group of patients who do not die within 2 years, NKp30 expression significantly increased at day 30 (Figure 3B). This increase remained significant until day 60. By contrast, in the group of patients who die within 2 years after diagnosis, no significant increase was evidenced compared to diagnosis (Figure 3B). We then analyzed survival stratified by NKp30 expression at day 30 in patients with intermediate cytogenetics. NKp30 status after complete remission was a better predictor of clinical outcome than NKp30 status at diagnosis, with higher HR for OS (HR = 0.20 (95%CI = [0.04–0.82], *P* = 0.025) and RFS (HR = 0.24 (95%CI = [0.07–0.81], *P* = 0.041) (Figure 3C and 3D, respectively).

### NKp30 downregulation appears early during NK maturation

**Figure 4:**
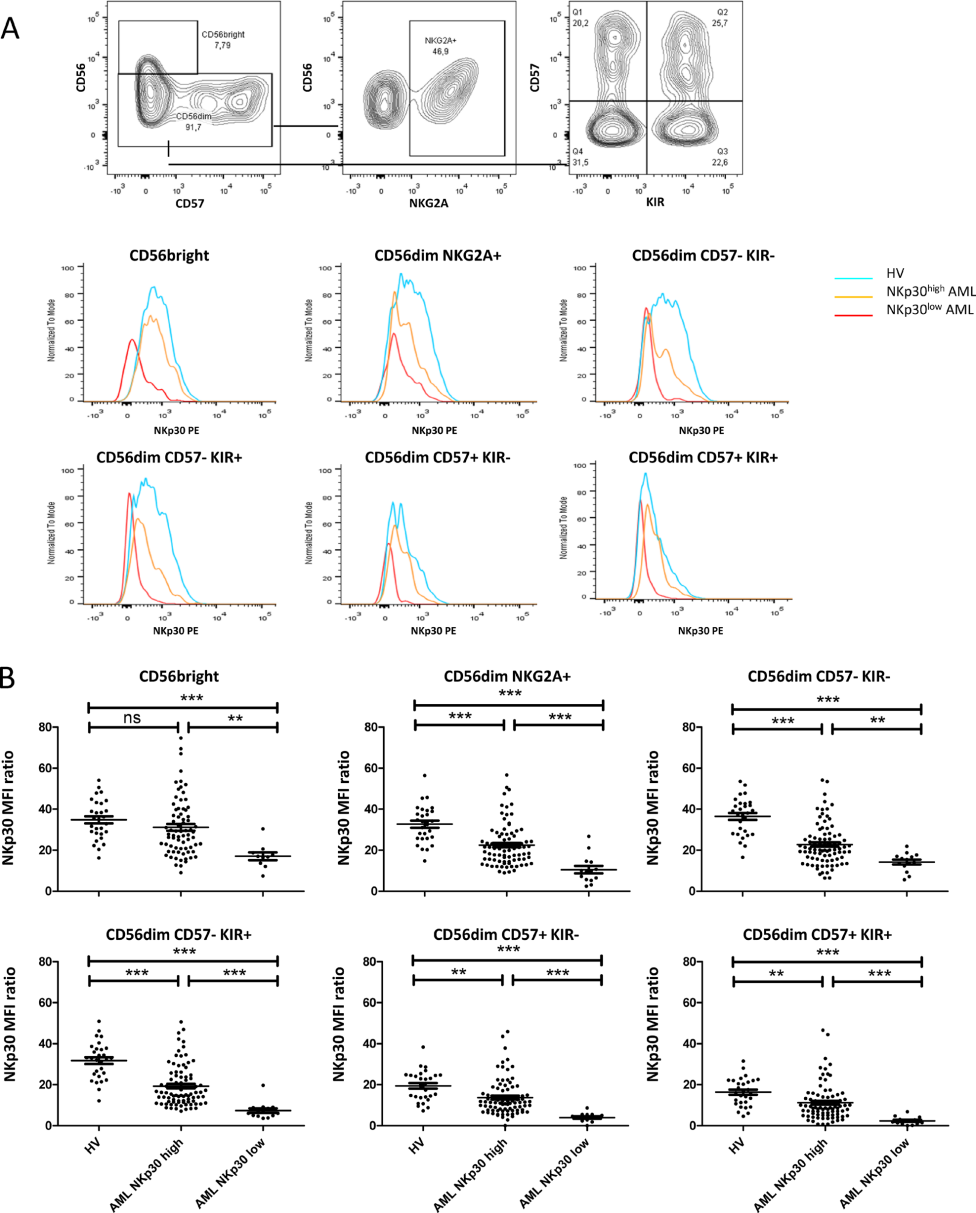
NKp30 down-regulation by NK subpopulations (**A**) PBMCs were labeled with anti-CD45, -CD56, -CD3 antibodies. Monocytes were identified using an exclusion gating strategy described in Supplementary Figure 2, and NK subsets were separated on CD56, NKG2A, KIR and CD57 expression. NKp30 expression on the different subsets of NK cells is displayed for a representative patient of each group (NKp30low or NKp30 high) and for a healthy volunteer. (**B**) Flow cytometry data from 101 AML patients and 29 HV were analyzed for NKp30 expression on each subsets of NK cells. Statistical analyses were performed using a Kruskal-Wallis test was used followed by a Dunn's pos*t*-test. HV: healthy volunteer. ns: non significant; *P < 0.05; **P < 0.01; ***P < 0.001

The mechanisms leading to low NKp30 expression in AML are poorly described. Notably, the kinetics of NKp30 down-regulation or blockade of expression during the different stages of NK maturation has not been described in human. To address this question, NKp30 expression was assessed by NK maturation subsets on 101 AML patients and 29 HV. NK cells maturation subtypes were defined according to CD56, CD57 and KIR (CD158a,h and CD158b1,b2,j) expression (Figure 4A). In patients with NKp30^low^ phenotype, NKp30 down-regulation was significant in all the clusters of NK cells, independent of the maturation stage (Figure 4B). Interestingly, NKp30 downregulation was observed at early stage of NK differentiation, notably in CD56^bright^ NK cells and in CD57^−^ NK cells, which are the clusters that express highest levels of NKp30 in healthy volunteers.

### NKp30 ligands

Expression of NKp30 ligand B7-H6 was assessed by flow cytometry on leukemic blasts of 39 patients from the GOELAMS cohort. B7-H6 was detected on 5% of leukemic blasts in 1 out of 39 patients (Supplementary Figure 3A). B7-H6 expression on leukemic blasts was not associated with NKp30^low^ phenotype in these patients.

We performed ELISA assays to detect soluble B7-H6 (sB7-H6) and soluble BAG6 (sBAG6) in plasma samples of AML patients at diagnosis (*N* = 17). NKp30 expression was assessed in paired samples of cryopreserved PBMCs, and analyzed regarding to the presence or the absence of detectable sB7-H6 and sBAG6. sB7-H6 was detectable in the serum of 4 out of 17 patients (mean ± SD = 2.1 mg/mL ± 6.4; range: 0 to 31 ng/mL). sBAG6 was detectable in the serum of 15 out of 17 patients (mean ± SD = 11.45 mg/mL ± 11.41; range: 0 to 39.3 ng/mL). NKp30 expression was not significantly associated with a decreased NKp30 expression in patients with detectable plasmatic sB7H6 or with the presence of BAG6 (Supplementary Figure 3B).

## DISCUSSION

In acute myeloid leukemia (AML), the current prognosis classification based on cytogenetics and genetic mutations defines 3 groups, i.e. good, adverse and intermediate prognosis, the latter being a group of patients for which clinical decision making is difficult with respect to post-remission therapy. A refined classification for this subgroup of patients is therefore required. We hypothesized that adverse clinical outcome may be explained by a failure of the immune system to control the disease. Accordingly, a better knowledge of the immune system's status may better classify patients especially in subgroups of patients with intermediate prognosis.

AML develops from somatically acquired mutations, some of which being the basis of prognostic classifications [7]. However, the microenvironment and in particular deficient immunity is deeply involved in tumor progression as well as resistance to chemotherapy and immunotherapy [12, 28–31]. This assumption is currently extensively explored in immunomonitoring studies in both solid tumors and hematologic malignancies, and provides consistent information for clinical outcome prediction [18, 20, 22, 32–35]. Standard patient classification can thus be improved or completed with extremely informative immune parameters, either used alone or through combinatorial approaches with immune signatures, as recently shown in solid tumors [32–34, 36].

AML is an archetypal model of immune-sensitive cancers, notably to Natural Killer (NK) cells [37–41]. NK cells are immune effectors that mediate cytotoxic effects against leukemic cells. NK-specific natural cytotoxicity receptors (NCR), including NKp30, are among the most important receptors that trigger specific cytolytic responses to tumor target cells [18, 42–45]. Several studies have reported NCR deficiencies in hematologic malignancies as well as in solid tumors, with significant impact on clinical outcome [18, 20–22] and control of the minimal residual disease [13].

In line, our group has demonstrated the importance of NK cell triggering receptor NKp30 for immune recognition and lysis of leukemic blasts, with significant impact on patient survival in an exploratory study involving 81 patients with AML [18]. However, this proof of concept lacked demonstration of clinical impact on relapse risk. In addition, a specific analysis in subgroups of patients with intermediate cytogenetics and ELN was warranted before any clinical applications. Therefore, the present study was designed to validate the prognostic significance of NKp30 on homogenous cohorts of patients in terms of age and chemotherapy, with a focus on intermediate-risk AML.

In our cohort, the prognostic value of NKp30 was significant in both OS and RFS in the group of 162 patients with intermediate cytogenetics. To date, most studies aiming at stratifying patients with AML, have focused on molecular genetic lesions, and significantly improved AML patient stratification [46–51]. Some markers such as FLT3-ITD, CEBPα and NPM1 [3, 5, 6, 49] are already used in clinical practice, and additional markers have been recently included in recommendations for prognostication. We assessed the prognostic significance of NKp30 in patients with intermediate ELN. We demonstrated that patients with intermediate ELN were still significantly stratified by NKp30 expression, with improved discrimination evidenced by an increase of the c-index compared to the cytogenetic and ELN classifications. In addition, we show that NKp30 is an independent predictor of clinical outcome in multivariate regression analyses even when accounting for the potential effects of allogeneic SCT, with a strong correlation between NKp30 status and RFS, OS and CIR.

Nonetheless, some patients with intermediate ELN and high NKp30 expression have an adverse clinical outcome. These false negative cases strongly suggest that additional parameters are involved in relapse. The immune landscape of tumors is obviously more complex, [52] and beside alterations of NCR, additional anomalies have been described on both NK cell [53–55] and T cell subsets [56, 57]. Clinical consequences of such immune profiles remain to be tested or validated. Yet, this appeals for integrated analysis of the immune responses in AML.

Since low NKp30 expression is associated with poor prognosis, restoring NKp30 on NK cells is a therapeutic strategy likely to improve clinical outcome in AML patients.

However, the mechanisms leading to low NKp30 expression in AML are not fully understood. In the present study, the low NKp30 expression observed in a fraction of patients is present at all maturation stages, with the extreme CD56^bright^ versus CD56^dim^ KIR^+^CD57^+^ NK cell population. As previously shown, CD57^+^ NK cells in HV display lower levels of NKp30 [58] compared to immature NK cells, and in AML patients we observed a lower expression in CD57^+^ NK cells in both group of patients (NKp30^high^ and NKp30^low^). These data suggests that NKp30 low expression in NKp30^low^ AML patient group is unlinked to NK cell maturation.

One of the hypotheses underlying the mechanisms involved in NKp30 down regulation in AML, a chronic exposure to Nkp30 ligands such as B7-H6 or BAG6 has been proposed and demonstrated in other cancers [59]. In our study, the expression of membrane-bound B7-H6 was found extremely weak and only in one patient and not associated with NKp30 downregulation. As a soluble form of B7H6 and BAG6 can be found in the serum, we evaluated whether the presence or absence of these ligands was associated with a lower membranous expression of NKp30. Our data revealed that soluble B7-H6 was detectable in 4 out of 20 patients but was not associated with NKp30 low expression. Although we could detect BAG6 in more patients, the presence of the ligands was not associated with a lower NKp30 expression. Therefore our data do not suggest that exposure to membrane-bound or soluble form of NKp30 ligands is involved in NKp30 down regulation in AML. Although these data should be considered as preliminary regarding the lower number of patients, the mechanisms of NKp30 low expression in AML remain elusive and warrant further studies.

An important question remains the best time point to evaluate NKp30 expression. To date, all studies assessing the prognostic value of NKp30 were performed at diagnosis. However, major variations of NKp30 expression have been described after induction chemotherapy [18, 60]. In the present study, NKp30 expression recovery after complete remission significantly stratifies patients with poor clinical outcome. Thus, NKp30 status appears as a relevant surrogate marker to monitor after complete remission.

Importantly, NKp30 status at diagnosis is assessable on peripheral blood cells by flow cytometry, a fast, cost-effective and reproducible method broadly used and fully validated. Its clinical applications have recently been further developed with the evaluation of minimal residual disease which implies sequential testing in the course of therapy [61]. Therefore this method can be readily included in the algorithm of treatment decision.

To conclude, our data demonstrates that NKp30 expression at diagnosis is an independent prognostic biomarker that refines classifications used in clinical practice. To our knowledge, NKp30 expression on NK cells is the first prognostic biomarker in intermediate risk AML assessable at diagnosis and based on an immune parameter. The present study provides bases for using NKp30 status as a prognostic biomarker for clinical decision-making regarding post-remission therapy. Moreover, our study provides a strong rationale to develop therapeutic strategies to maintaining high levels of NKp30 expression to improve clinical outcome after complete remission. Complementary studies are required to further explore the mechanisms of NKp30 down-regulation for the development of therapeutic strategies for patients with defective NK cells.

## MATERIALS AND METHODS

### Patients and study design

Baseline NKp30 expression on NK cells at diagnosis was assessed in a total of 201 patients. Two cohorts of patients were merged in the present study. The Paoli Calmettes Institute (IPC) prospective cohort included 115 patients with newly diagnosed non-acute promyelocytic leukemia (APL) AML admitted between November 2007 and November 2012, aged 18 to 65 years (mean ± SD = 47.1 ± 10.6) and treated with conventional 3+7 induction chemotherapy as previously described [23]. The Groupe Ouest Est d'Etude des Leucémies Aiguës et autres Maladies du Sang (GOELAMS) cohort included 86 patients from the LAM2006IR prospective multicenter randomized trial, included between November 2007 and April 2012 (NCT00860639), aged 18 to 65 years (mean ± SD = 46.3 ± 11.0). All patients had previously untreated AML with intermediate cytogenetics. Patients received conventional 3+7 induction chemotherapy with or without the addition of Gemtuzumab Ozogamicin [24]. Patients with APL AML and patients above 66 years were excluded.

### Ethics statement

All participants gave written informed consent in accordance with the Declaration of Helsinki. The entire research procedure was approved by the ethical review boards from the IPC and the GOELAMS.

### Clinical samples

Fresh total peripheral blood samples (IPC cohort) or peripheral blood mononuclear cells (PBMC) cryopreserved in 90%FCS/10%DMSO (GOELAMS cohort) were obtained from randomly selected patients before induction chemotherapy and from age-matched healthy volunteers. For the GOELAMS cohort, handling, conditioning and storing of samples were performed by the FILOtheque AML (N° BB-0033–00073), tumor bank of the FILO group, Cochin hospital, Paris.

### Flow cytometry analysis

A FACS Canto II (IPC cohort) and a LSR Fortessa (GOELAMS cohort) (BD Biosciences, San Jose, CA), and FACS Diva Software (BD Biosciences) and FlowJo Software (Treestar, Inc., San Carlos, CA) were used for flow cytometry. NK cells from whole blood EDTA or frozen PBMC were immunostained with antibodies listed in Supplementary Table 1. Red blood cells were lysed with BD FACS Lysing solution (BD Biosciences) before acquisition. The NKp30 mean fluorescence intensity (MFI) ratio (NKp30 MFI/isotype control MFI, referred to as rMFI) was calculated for each patient. NKp30 expression was assessed at diagnosis and after complete remission, at day 30, 60 and 90 after the last induction chemotherapy. When material was available, leukemic blasts were stained with antibodies listed in Supplementary Table 2. Analyses were performed in the Biopathology department and on the IPC Immunomonitoring platform. Samples from the IPC cohort and from the GOELAMS cohort were analyzed blinded to the study endpoint.

### Threshold determination

**Figure 5:**
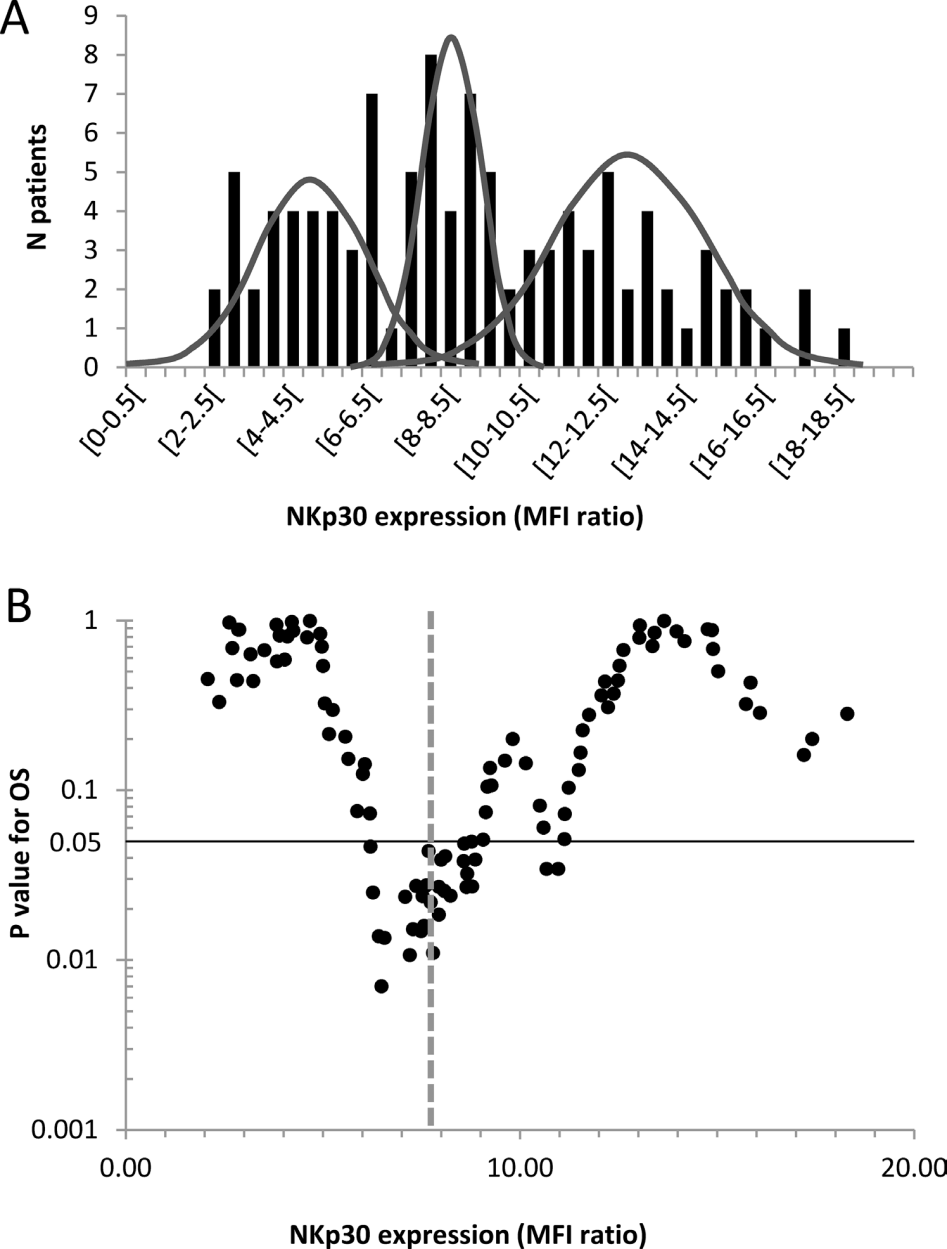
Threshold determination for NKp30 expression on NK cells (IPC prospective cohort) (**A**) Distribution histograms of NKp30 mean fluorescence intensity (MFI) ratio (NKp30 MFI / isotype control MFI) in patients with AML at diagnosis. The curves are estimates of population density distribution. (**B**) The volcano plot shows log of *P* value for overall survival according to threshold for NKp30 expression at diagnosis. The dashed line represents the threshold used in the rest of the study. The black line represents the limit of statistical significance (*P* < 0.05). The normality of distributions were evaluated with a d'Agostino-Pearson normality test and a Kernel density estimation.

Patients were classified into two groups according to NKp30 rMFI. The dichotomy between NKp30^low^ and NKp30^high^ patients was based on dispersion criteria of the IPC prospective cohort. Figure 5A displays inter-individual variability of NKp30 expression in AML patients. The distribution of NKp30 expression was a juxtaposition of three Gaussian distributions (d'Agostino-Pearson normality test and Kernel density estimation). The intersection between the first and the second peak was NKp30 rMFI = 7.8 (Figure 5A). All the possible thresholds were tested in the range of NKp30 expression for overall survival (OS) (Figure 5B). The threshold based on dispersion criteria was discriminant for survival analyses.

For samples from the GOELAMS cohort, analyses were performed on frozen PBMCs on a different cytometer. Paired samples were analyzed on both cytometers (fresh total blood on a CantoII and paired frozen PBMCs on a LSR Fortessa). The correlation between both methods was high (r = 0.90) and no correction factor was applied to the measurements of NKp30 expression in the GOELAMS cohort. For the rest of the study, patients from both cohorts were classified into 2 distinct subgroups (NKp30^high^ and NKp30^low^ phenotype) for survival analyses according to this threshold.

For patients assessable for NKp30 expression after complete remission, a second threshold was determined according to the methodology described above. The intersection between the 2 Gaussian distributions was 18.6 (Supplementary Figure 1).

### Soluble B7-H6 (sB7-H6) and BAG6 (sBAG6) detection by ELISA

ELISA were performed as previously described [25]. Briefly, 96-well plates were coated with 5μg/mL mouse anti-B7-H6 mAb (clone 5.51.18) or anti-BAG6 mAb. B7-H6-Fc and BAG6 fusion protein were used as standard. The standard and the samples were added at 100μL/well, and the plates were incubated overnight at 4°C. After incubation, plates were washed and B7-H6 protein was detected with the biotinylated rabbit anti- B7-H6 796 antibody at a final concentration of 1 mg/mL in PBS/ 0.05% Tween20 for 2 hours at room temperature. After washing, streptavidin-HRP (1:200; R&D) was added for 30 minutes at room temperature. Subsequently, plates were washed and developed using 3,3′,5,5′-tetramethylbenzidine (TMB) Liquid Substrate System (Sigma). The absorbance was measured at 450 nm.

### End points and statistical analysis

Statistical analyses were carried out using SPSS (SPSS software, Chicago, IL), Graph Pad Prism (Graph Pad Software, San Diego, CA) and R software (www.r-project.org). The limit of significance was set at *P* < 0.05. The X^2^, Fisher's exact test and t test were used to compare baseline variables among patients with high or low NKp30 phenotype. For multiple comparisons, a Kruskal-Wallis test was used followed by a Dunn's pos*t*-test. OS was defined as the time from diagnosis until death from any cause, and relapse-free survival (RFS) as the time between induction and relapse or death, whatever occurred first. Data were censored at the date of allo-SCT in 1^st^ complete remission. Patients without allo-SCT in CR1 and without an event (death or relapse) were censored at the time of their last follow-up. Survival times were estimated by Kaplan-Meier method and compared using the log-rank test. A multivariate Cox regression model was used to assess the predictive value of NKp30 expression while adjusting for other prognostic factors (age at diagnosis, disease status, ELN, leukocytosis, and allo-SCT as a time-dependent covariate). Cumulative incidence of relapse (CIR) was calculated using cumulative incidence estimates to accommodate competing risks, using a Cox regression model, while adjusting for prognostic factors cited above. All the subgroup analyses were defined *a priori*.

Model discrimination was assessed by the c-index after exclusion of patients with allo-SCT in first complete remission [26]. This study conformed to the recommendations for tumor marker prognostic studies (REMARK) [27].

## SUPPLEMENTARY MATERIALS TABLES AND FIGURES



## References

[1] Grimwade D, Walker H, Oliver F, Wheatley K, Harrison C, Harrison G, Rees J, Hann I, Stevens R, Burnett A (1998). The importance of diagnostic cytogenetics on outcome in AML: analysis of 1,612 patients entered into the MRC AML 10 trial. Blood.

[2] Slovak ML, Kopecky KJ, Cassileth PA, Harrington DH, Theil KS, Mohamed A, Paietta E, Willman CL, Head DR, Rowe JM (2000). Karyotypic analysis predicts outcome of preremission and postremission therapy in adult acute myeloid leukemia: a Southwest Oncology Group/Eastern Cooperative Oncology Group Study. Blood.

[3] Dohner H, Estey EH, Amadori S, Appelbaum FR, Buchner T, Burnett AK, Dombret H, Fenaux P, Grimwade D, Larson RA, Lo-Coco F, Naoe T, Niederwieser D (2010). Diagnosis and management of acute myeloid leukemia in adults: recommendations from an international expert panel, on behalf of the European LeukemiaNet. Blood.

[4] Stelljes M, Krug U, Beelen DW, Braess J, Sauerland MC, Heinecke A, Ligges S, Sauer T, Tschanter P, Thoennissen GB, Berning B, Kolb HJ, Reichle A (2014). Allogeneic transplantation versus chemotherapy as postremission therapy for acute myeloid leukemia: a prospective matched pairs analysis. J Clin Oncol.

[5] Grimwade D, Hills RK, Moorman AV, Walker H, Chatters S, Goldstone AH, Wheatley K, Harrison CJ, Burnett AK, Group NCRIALW (2010). Refinement of cytogenetic classification in acute myeloid leukemia: determination of prognostic significance of rare recurring chromosomal abnormalities among 5876 younger adult patients treated in the United Kingdom Medical Research Council trials. Blood.

[6] Patel JP, Gönen M, Figueroa ME, Fernandez H, Sun Z, Racevskis J, Van Vlierberghe P, Dolgalev I, Thomas S, Aminova O (2012). Prognostic relevance of integrated genetic profiling in acute myeloid leukemia. New England Journal of Medicine.

[7] Döhner H, Estey E, Grimwade D, Amadori S, Appelbaum FR, Büchner T, Dombret H, Ebert BL, Fenaux P, Larson RA, Levine RL, Lo-Coco F, Naoe T (2017). Diagnosis and management of AML in adults: 2017 ELN recommendations from an international expert panel. Blood.

[8] Vivier E, Raulet DH, Moretta A, Caligiuri MA, Zitvogel L, Lanier LL, Yokoyama WM, Ugolini S (2011). Innate or adaptive immunity? The example of natural killer cells. Science.

[9] Vivier E, Ugolini S, Blaise D, Chabannon C, Brossay L (2012). Targeting natural killer cells and natural killer T cells in cancer. Nature Reviews Immunology.

[10] Raulet DH (2004). Interplay of natural killer cells and their receptors with the adaptive immune response. Nat Immunol.

[11] Menard C, Blay JY, Borg C, Michiels S, Ghiringhelli F, Robert C, Nonn C, Chaput N, Taieb J, Delahaye NF, Flament C, Emile JF, Le Cesne A (2009). Natural killer cell IFN-gamma levels predict long-term survival with imatinib mesylate therapy in gastrointestinal stromal tumor-bearing patients. Cancer Res.

[12] Zitvogel L, Galluzzi L, Smyth MJ, Kroemer G (2013). Mechanism of action of conventional and targeted anticancer therapies: reinstating immunosurveillance. Immunity.

[13] Sullivan EM, Jeha S, Kang G, Cheng C, Rooney B, Holladay M, Bari R, Schell S, Tuggle M, Pui CH (2014). NK cell genotype and phenotype at diagnosis of acute lymphoblastic leukemia correlate with postinduction residual disease. Clinical Cancer Research.

[14] Cooley S, Weisdorf DJ, Guethlein LA, Klein JP, Wang T, Le CT, Marsh SG, Geraghty D, Spellman S, Haagenson MD, Ladner M, Trachtenberg E, Parham P (2010). Donor selection for natural killer cell receptor genes leads to superior survival after unrelated transplantation for acute myelogenous leukemia. Blood.

[15] Horowitz MM, Gale RP, Sondel PM, Goldman JM, Kersey J, Kolb HJ, Rimm AA, Ringden O, Rozman C, Speck B (1990). Graft-versus-leukemia reactions after bone marrow transplantation. Blood.

[16] Norell H, Moretta A, Silva-Santos B, Moretta L At the Bench: Preclinical rationale for exploiting NK cells and gammadelta T lymphocytes for the treatment of high-risk leukemias. J Leukoc Biol.

[17] Ruggeri L, Mancusi A, Capanni M, Urbani E, Carotti A, Aloisi T, Stern M, Pende D, Perruccio K, Burchielli E, Topini F, Bianchi E, Aversa F (2007). Donor natural killer cell allorecognition of missing self in haploidentical hematopoietic transplantation for acute myeloid leukemia: challenging its predictive value. Blood.

[18] Fauriat C, Just-Landi S, Mallet F, Arnoulet C, Sainty D, Olive D, Costello RT (2007). Deficient expression of NCR in NK cells from acute myeloid leukemia: Evolution during leukemia treatment and impact of leukemia cells in NCRdull phenotype induction. Blood.

[19] Pende D, Parolini S, Pessino A, Sivori S, Augugliaro R, Morelli L, Marcenaro E, Accame L, Malaspina A, Biassoni R, Bottino C, Moretta L, Moretta A (1999). Identification and molecular characterization of NKp30, a novel triggering receptor involved in natural cytotoxicity mediated by human natural killer cells. J Exp Med.

[20] Delahaye NF, Rusakiewicz S, Martins I, Menard C, Roux S, Lyonnet L, Paul P, Sarabi M, Chaput N, Semeraro M, Minard-Colin V, Poirier-Colame V, Chaba K (2011). Alternatively spliced NKp30 isoforms affect the prognosis of gastrointestinal stromal tumors. Nat Med.

[21] Khaznadar Z, Boissel N, Agaugue S, Henry G, Cheok M, Vignon M, Geromin D, Cayuela JM, Castaigne S, Pautas C, Raffoux E, Lachuer J, Sigaux F (2015). Defective NK Cells in Acute Myeloid Leukemia Patients at Diagnosis Are Associated with Blast Transcriptional Signatures of Immune Evasion. J Immunol.

[22] Pasero C, Gravis G, Granjeaud S, Guerin M, Thomassin-Piana J, Rocchi P, Salem N, Walz J, Moretta A, Olive D (2015). Highly effective NK cells are associated with good prognosis in patients with metastatic prostate cancer. Oncotarget.

[23] Devillier R, Gelsi-Boyer V, Murati A, Prebet T, Rey J, Etienne A, D'Incan E, Charbonnier A, Blaise D, Mozziconacci MJ (2015). Prognostic significance of myelodysplasia-related changes according to the WHO classification among ELN-intermediate-risk AML patients. American journal of hematology.

[24] Hills RK, Castaigne S, Appelbaum FR, Delaunay J, Petersdorf S, Othus M, Estey EH, Dombret H, Chevret S, Ifrah N (2014). Addition of gemtuzumab ozogamicin to induction chemotherapy in adult patients with acute myeloid leukaemia: a meta-analysis of individual patient data from randomised controlled trials. The Lancet Oncology.

[25] Schlecker E, Fiegler N, Arnold A, Altevogt P, Rose-John S, Moldenhauer G, Sucker A, Paschen A, von Strandmann EP, Textor S (2014). Metalloprotease-Mediated Tumor Cell Shedding of B7-H6, the Ligand of the Natural Killer Cell–Activating Receptor NKp30. Cancer research.

[26] Harrell FE, Lee KL, Mark DB (1996). Tutorial in biostatistics multivariable prognostic models: issues in developing models, evaluating assumptions and adequacy, and measuring and reducing errors. Statistics in medicine.

[27] McShane LM, Altman DG, Sauerbrei W, Taube SE, Gion M, Clark GM (2005). Statistics Subcommittee of the NCI-EORTC Working Group on Cancer Diagnostics. Reporting recommendations for tumor marker prognostic studies (REMARK). J Natl Cancer Inst.

[28] Zitvogel L, Kepp O, Kroemer G (2011). Immune parameters affecting the efficacy of chemotherapeutic regimens. Nat Rev Clin Oncol.

[29] Hanahan D, Weinberg RA (2011). Hallmarks of cancer: the next generation. Cell.

[30] Martner A, Rydström A, Riise RE, Aurelius J, Brune M, Foà R, Hellstrand K, Thorén FB (2015). NK cell expression of natural cytotoxicity receptors may determine relapse risk in older AML patients undergoing immunotherapy for remission maintenance. Oncotarget.

[31] Martner A, Rydström A, Riise RE, Aurelius J, Anderson H, Brune M, Foà R, Hellstrand K, Thorén FB (2016). Role of natural killer cell subsets and natural cytotoxicity receptors for the outcome of immunotherapy in acute myeloid leukemia. Oncoimmunology.

[32] Galon J, Costes A, Sanchez-Cabo F, Kirilovsky A, Mlecnik B, Lagorce-Pagès C, Tosolini M, Camus M, Berger A, Wind P (2006). Type, density, and location of immune cells within human colorectal tumors predict clinical outcome. Science.

[33] Mlecnik B, Bindea G, Kirilovsky A, Angell HK, Obenauf AC, Tosolini M, Church SE, Maby P, Vasaturo A, Angelova M (2016). The tumor microenvironment and Immunoscore are critical determinants of dissemination to distant metastasis. Sci Transl Med.

[34] Mlecnik B, Tosolini M, Kirilovsky A, Berger A, Bindea G, Meatchi T, Bruneval P, Trajanoski Z, Fridman WH, Pagès F (2011). Histopathologic-based prognostic factors of colorectal cancers are associated with the state of the local immune reaction. Journal of Clinical Oncology.

[35] Donskov F, von der Maase H (2006). Impact of immune parameters on long-term survival in metastatic renal cell carcinoma. Journal of Clinical Oncology.

[36] Mlecnik B, Bindea G, Angell HK, Maby P, Angelova M, Tougeron D, Church SE, Lafontaine L, Fischer M, Fredriksen T (2016). Integrative analyses of colorectal cancer show immunoscore is a stronger predictor of patient survival than microsatellite instability. Immunity.

[37] Parameswaran R, Ramakrishnan P, Moreton SA, Xia Z, Hou Y, Lee DA, Gupta K, Beck RC, Wald DN (2016). Repression of GSK3 restores NK cell cytotoxicity in AML patients. Nature communications.

[38] Bachanova V, Cooley S, Defor TE, Verneris MR, Zhang B, McKenna DH, Curtsinger J, Panoskaltsis-Mortari A, Lewis D, Hippen K (2014). Clearance of acute myeloid leukemia by haploidentical natural killer cells is improved using IL-2 diphtheria toxin fusion protein. Blood.

[39] Miller JS, Soignier Y, Panoskaltsis-Mortari A, McNearney SA, Yun GH, Fautsch SK, McKenna D, Le C, Defor TE, Burns LJ (2005). Successful adoptive transfer and in vivo expansion of human haploidentical NK cells in patients with cancer. Blood.

[40] Ruggeri L, Mancusi A, Perruccio K, Burchielli E, Martelli MF, Velardi A (2005). Natural killer cell alloreactivity for leukemia therapy. Journal of Immunotherapy.

[41] Vey N, Bourhis JH, Boissel N, Bordessoule D, Prebet T, Charbonnier A, Etienne A, Andre P, Romagne F, Benson D, Dombret H, Olive D (2012). A phase 1 trial of the anti-inhibitory KIR mAb IPH2101 for AML in complete remission. Blood.

[42] Fauriat C, Just-Landi S, Mallet F, Arnoulet C, Sainty D, Olive D, Costello RT (2007). Deficient expression of NCR in NK cells from acute myeloid leukemia: Evolution during leukemia treatment and impact of leukemia cells in NCRdull phenotype induction. Blood.

[43] Fauriat C, Marcenaro E, Sivori S, Rey J, Gastaut JA, Moretta A, Olive D, Costello RT (2003). Natural killer cell-triggering receptors in patients with acute leukaemia. Leuk Lymphoma.

[44] Vossen MT, Matmati M, Hertoghs KM, Baars PA, Gent MR, Leclercq G, Hamann J, Kuijpers TW, van Lier RA (2008). CD27 defines phenotypically and functionally different human NK cell subsets. The Journal of Immunology.

[45] Dulphy N, Chrétien AS, Khaznadar Z, Fauriat C, Nanbakhsh A, Caignard A, Chouaib S, Olive D, Toubert A (2016). Underground Adaptation to a Hostile environment: Acute Myeloid Leukemia vs. Natural Killer Cells. Frontiers in Immunology.

[46] Arber DA, Orazi A, Hasserjian R, Thiele J, Borowitz MJ, Le Beau MM, Bloomfield CD, Cazzola M, Vardiman JW (2016). The 2016 revision to the World Health Organization (WHO) classification of myeloid neoplasms and acute leukemia. Blood.

[47] Marcucci G, Haferlach T, Döhner H (2011). Molecular genetics of adult acute myeloid leukemia: prognostic and therapeutic implications. J Clin Oncol.

[48] Meyer SC, Levine RL (2014). Translational implications of somatic genomics in acute myeloid leukaemia. The Lancet Oncology.

[49] Döhner H, Weisdorf DJ, Bloomfield CD (2015). Acute myeloid leukemia. New England Journal of Medicine.

[50] Papaemmanuil E, Gerstung M, Bullinger L, Gaidzik VI, Paschka P, Roberts ND, Potter NE, Heuser M, Thol F, Bolli N (2016). Genomic Classification and Prognosis in Acute Myeloid Leukemia. New England Journal of Medicine.

[51] Thol F, Damm F, Lüdeking A, Winschel C, Wagner K, Morgan M, Yun H, Göhring G, Schlegelberger B, Hoelzer D (2011). Incidence and prognostic influence of DNMT3A mutations in acute myeloid leukemia. Journal of Clinical Oncology.

[52] Gentles AJ, Newman AM, Liu CL, Bratman SV, Feng W, Kim D, Nair VS, Xu Y, Khuong A, Hoang CD (2015). The prognostic landscape of genes and infiltrating immune cells across human cancers. Nature medicine.

[53] Chretien AS, Granjeaud S, Gondois-Rey F, Harbi S, Orlanducci F, Blaise D, Vey N, Arnoulet C, Fauriat C, Olive D (2015). Increased NK cell maturation in patients with Acute Myeloid Leukemia. Front Immunol.

[54] Stringaris K, Sekine T, Khoder A, Alsuliman A, Razzaghi B, Sargeant R, Pavlu J, Brisley G, de Lavallade H, Sarvaria A (2014). Leukemia-induced phenotypic and functional defects in natural killer cells predict failure to achieve remission in acute myeloid leukemia. Haematologica.

[55] Sanchez-Correa B, Campos C, Pera A, Bergua JM, Arcos MJ, Banas H, Casado JG, Morgado S, Duran E, Solana R, Tarazona R (2016). Natural killer cell immunosenescence in acute myeloid leukaemia patients: new targets for immunotherapeutic strategies?. Cancer Immunol Immunother.

[56] Le Dieu R, Taussig DC, Ramsay AG, Mitter R, Miraki-Moud F, Fatah R, Lee AM, Lister TA, Gribben JG (2009). Peripheral blood T cells in acute myeloid leukemia (AML) patients at diagnosis have abnormal phenotype and genotype and form defective immune synapses with AML blasts. Blood.

[57] Wetzler M, Baer M, Stewart S, Donohue K, Ford L, Stewart C, Repasky E, Ferrone S (2001). HLA class I antigen cell surface expression is preserved on acute myeloid leukemia blasts at diagnosis and at relapse. Leukemia.

[58] Bjorkstrom NK, Riese P, Heuts F, Andersson S, Fauriat C, Ivarsson MA, Bjorklund AT, Flodstrom-Tullberg M, Michaelsson J, Rottenberg ME, Guzman CA, Ljunggren HG, Malmberg KJ (2010). Expression patterns of NKG2A, KIR, and CD57 define a process of CD56dim NK-cell differentiation uncoupled from NK-cell education. Blood.

[59] Semeraro M, Rusakiewicz S, Minard-Colin V, Delahaye NF, Enot D, Vély F, Marabelle A, Papoular B, Piperoglou C, Ponzoni M (2015). Clinical impact of the NKp30/B7-H6 axis in high-risk neuroblastoma patients. Science translational medicine.

[60] Rey J, Anfossi N, Andre P, Boher JM, Orlanducci F, Breso V, Perri V, Prebet T, Charbonnier A, Perrot E (2011). Natural killer cells recovery after consolidation chemotherapy in elderly patients with acute myeloid leukemia (AML). Blood.

[61] Terwijn M, van Putten WL, Kelder A, van der Velden VH, Brooimans RA, Pabst T, Maertens J, Boeckx N, de Greef GE, Valk PJ (2013). High prognostic impact of flow cytometric minimal residual disease detection in acute myeloid leukemia: data from the HOVON/SAKK AML 42A study. Journal of Clinical Oncology.

