# Correction: NKp30 expression is a prognostic immune biomarker for stratification of patients with intermediate-risk acute myeloid leukemia

**DOI:** 10.18632/oncotarget.27198

**Published:** 2019-09-10

**Authors:** Anne-Sophie Chretien, Cyril Fauriat, Florence Orlanducci, Jerome Rey, Gaelle Bouvier Borg, Emmanuel Gautherot, Samuel Granjeaud, Clemence Demerle, Jean-François Hamel, Adelheid Cerwenka, Elke Pogge von Strandmann, Norbert Ifrah, Catherine Lacombe, Pascale Cornillet-Lefebvre, Jacques Delaunay, Antoine Toubert, Christine Arnoulet, Norbert Vey, Daniel Olive

**Affiliations:** ^1^ Team Immunity and Cancer, Centre de Recherche en Cancérologie de Marseille (CRCM), Inserm, U1068, CNRS, UMR7258, Institut Paoli-Calmettes, Aix-Marseille University, UM 105, Marseille, France; ^2^ Immunomonitoring Platform, Institut Paoli-Calmettes, Marseille, France; ^3^ Hematology Department, Centre de Recherche en Cancérologie de Marseille (CRCM), Inserm, U1068, CNRS, UMR7258, Institut Paoli-Calmettes, Aix-Marseille University, UM 105, Marseille, France; ^4^ Beckman Coulter Immunotech, Marseille, France; ^5^ Systems Biology Platform, Centre de Recherche en Cancérologie de Marseille (CRCM), Inserm, U1068, CNRS, UMR7258, Institut Paoli-Calmettes, Aix-Marseille University, UM 105, Marseille, France; ^6^ Biostatistics and Methodology Department, CHU Angers, Angers, France; ^7^ Innate Immunity Group, German Cancer Research Center, Heidelberg, Germany; ^8^ Department I of Internal Medicine, University Hospital of Cologne, Cologne, Germany; ^9^ Hematology Department, CHU Angers, Angers, France; ^10^ GOELAMStheque, FILO French Innovative Leukemia Organization, Cochin Hospital, APHP, Paris, France; ^11^ Laboratoire d’Hématologie, Centre Hospitalier Universitaire de Reims, Reims, France; ^12^ Service d’Hématologie, Centre Catherine de Sienne, Nantes, France; ^13^ INSERM UMRS-1160, Univ Paris Diderot, Sorbonne Paris Cité, Institut Universitaire d’Hématologie, Immunology and Histocompatibility Department, Hôpital Saint-Louis, APHP, Paris, France; ^14^ Biopathology Department, Institut Paoli Calmettes, Marseille, France; ^15^ Clinic for Hematology, Oncology and Immunology, Experimental Tumor Research, Center for Tumor Biology and Immunology, Philipps University, Marburg, Germany

**This article has been corrected:** Additional information has been added to the Funding section. The complete and updated Funding information is shown below:

## FUNDING

This work has been financially supported by the INCa, the SIRIC Marseille (grant INCa-DGOS-INSERM 6038), the Cancéropôle PACA (grants K_CyTOF 2014 and AML_CyTOF 2016 for J.A.N.), the GS IBiSA and the Agence Nationale de la Recherche (PHENOMIN project for MM HL and EG). The team “Immunity and Cancer” was labeled “Equipe FRM DEQ 201 40329534” (for D.O.). D.O. is a Senior Scholar of the Institut Universitaire de France. All antibodies used were kindly provided by Beckman-Coulter, Marseille, France. Beckman Coulter and the Beckman Coulter product and service marks mentioned herein are trademarks or registered trademarks of Beckman Coulter, Inc. in the United States and other countries. All other trademarks are the property of their respective owners. A part of this study was funded by a grant from the Wilhelm Sander Stiftung (2015.145.1) to EPvS.

Original article: Oncotarget. 2017; 8:49548–49563. 49548-49563. https://doi.org/10.18632/oncotarget.17747

