# Supplementary material for: NKp30 expression is a prognostic immune biomarker for stratification of patients with intermediate-risk acute myeloid leukemia
Source: Oncotarget. 2017 May 10;8(30):49548–63. doi: 10.18632/oncotarget.17747 (PMC5564787; doi:10.18632/oncotarget.17747)
Supplement: Supplementary file 1 [file oncotarget-08-49548-s001.pdf]

NKp30 expression is a prognostic immune biomarker for stratification of patients with intermediate-risk acute myeloid leukemia

Supplementary Materials

Supplementary Table 1: Immunostaining of NK cells

	IPC cohort	GOELAMS cohort	Clone	Company
CD45	APC	KO	J.33	Beckman-Coulter
CD3	FITC	ECD	UCHT1	Beckman-Coulter
CD56	PC7	APC	NKH-1	Beckman-Coulter
NKp30	PE	PE	Z25	Beckman-Coulter
CD158a,h	-	PC7	EB6B	Beckman-Coulter
CD158b1/b2/j	-	PC7	GL183	Beckman-Coulter
CD57	-	Pacific Blue	NC1	Beckman-Coulter
NKG2A	-	APC	Z199.1	Beckman-Coulter
Vivid	-	LIVE/DEAD® Near IR	-	Thermo Fisher Scientific
IgG Isotype	PE	PE	-	Beckman-Coulter

Abbreviations: APC: allophycocyanin; ECD: Phycoerythrin-Texas Red-x™*; FITC: fluorescein isothiocyanate; IR: infra-red; KO: Krome OrangeTM*; PC5: Phycoerythrin-Cyanine 5; PC7: Phycoerythrin-Cyanine 7 ; PE : Phycoerythrin.
* Krome Orange and Phycoerythrin-Texas Red-x are registered trademark of Beckman Coulter, Inc., in the United States and other countries

Supplementary Table 2: Immunostaining of leukemic blasts

Antigen	Fluorochrome	Clone	Company
CD45	KO	J.33	Beckman-Coulter
CD33	PC7	D3HL60.251	Beckman-Coulter
CD34	PC7	581	Beckman-Coulter
CD13	PC7	Immu103.44	Beckman-Coulter
CD15	PC7	HI98	BD Biosciences
CD14	APC H7	MϕP9	BD Biosciences
Live/Dead®	Near IR	-	Thermo Fisher Scientific
B7H6	Purified (goat)	5.51.18	Kind gift of Pr. Cerwenka
Anti-goat	PE	# IM0855	Beckman-Coulter

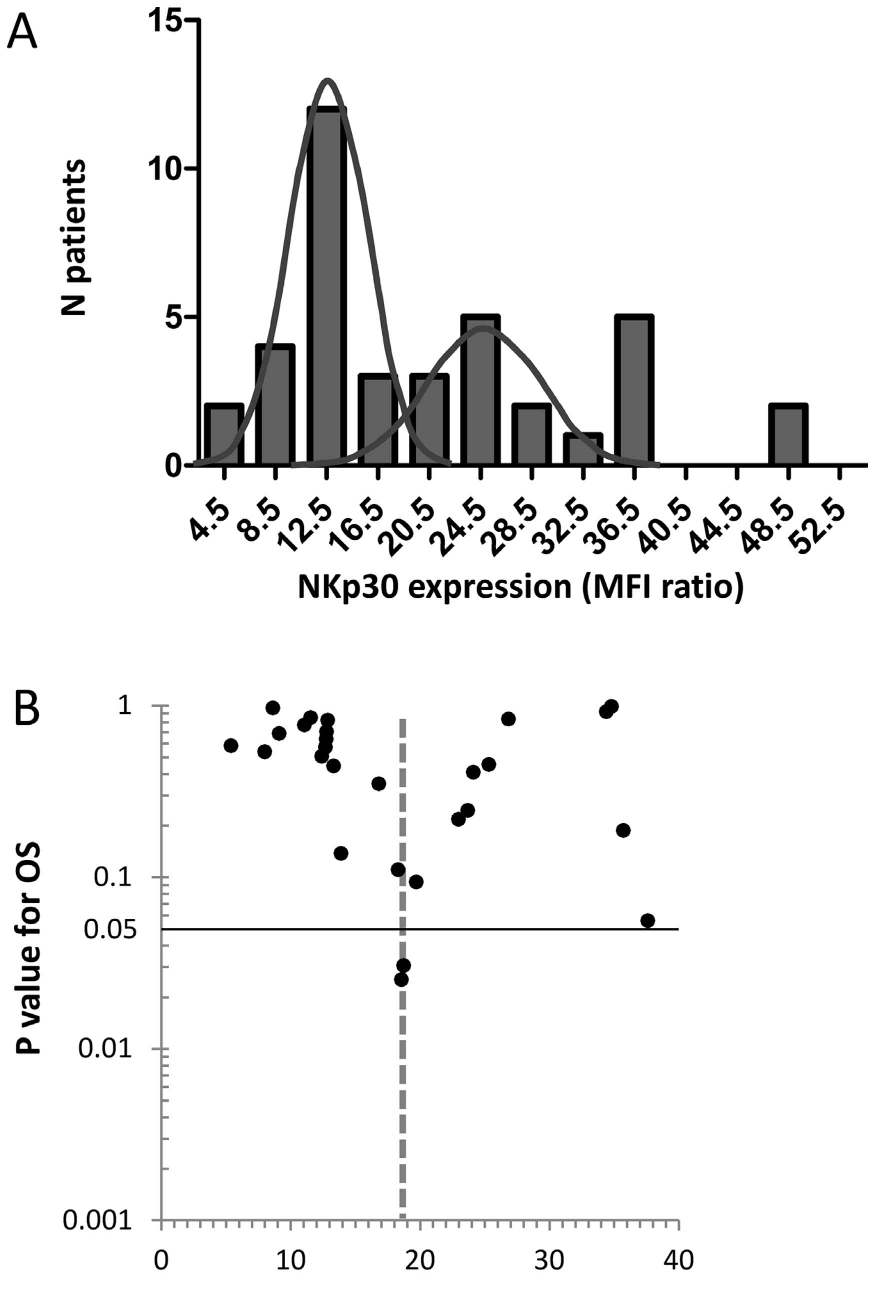

Supplementary Figure 1: Threshold determination for NKp30 expression on NK cells after complete remission (IPC prospective cohort). Distribution histograms of NKp30 mean fluorescence intensity (MFI) ratio (NKp30 MFI / isotype control MFI) in patients with AML at complete remission. The curves are estimates of population density distribution. The dashed line represents the threshold used in the rest of the study. The black line represents the limit of statistical significance ($P < 0.05$). The normality of distributions were evaluated with a d'Agostino-Pearson normality test.

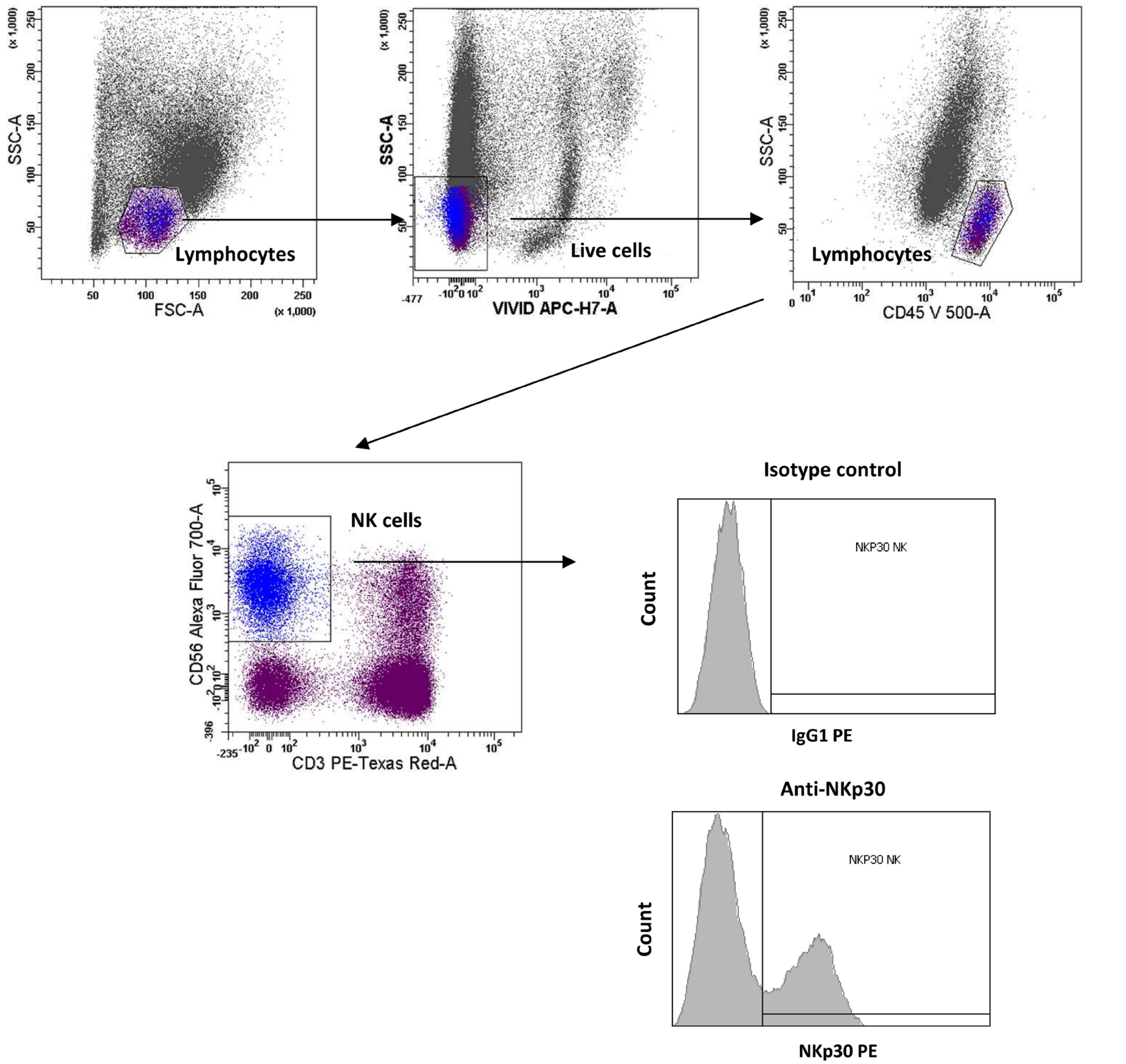

Supplementary Figure 2: Exemple of gating strategy for NK cells identification and NKp30 MFI ratio determination from of an AML patient at diagnosis.

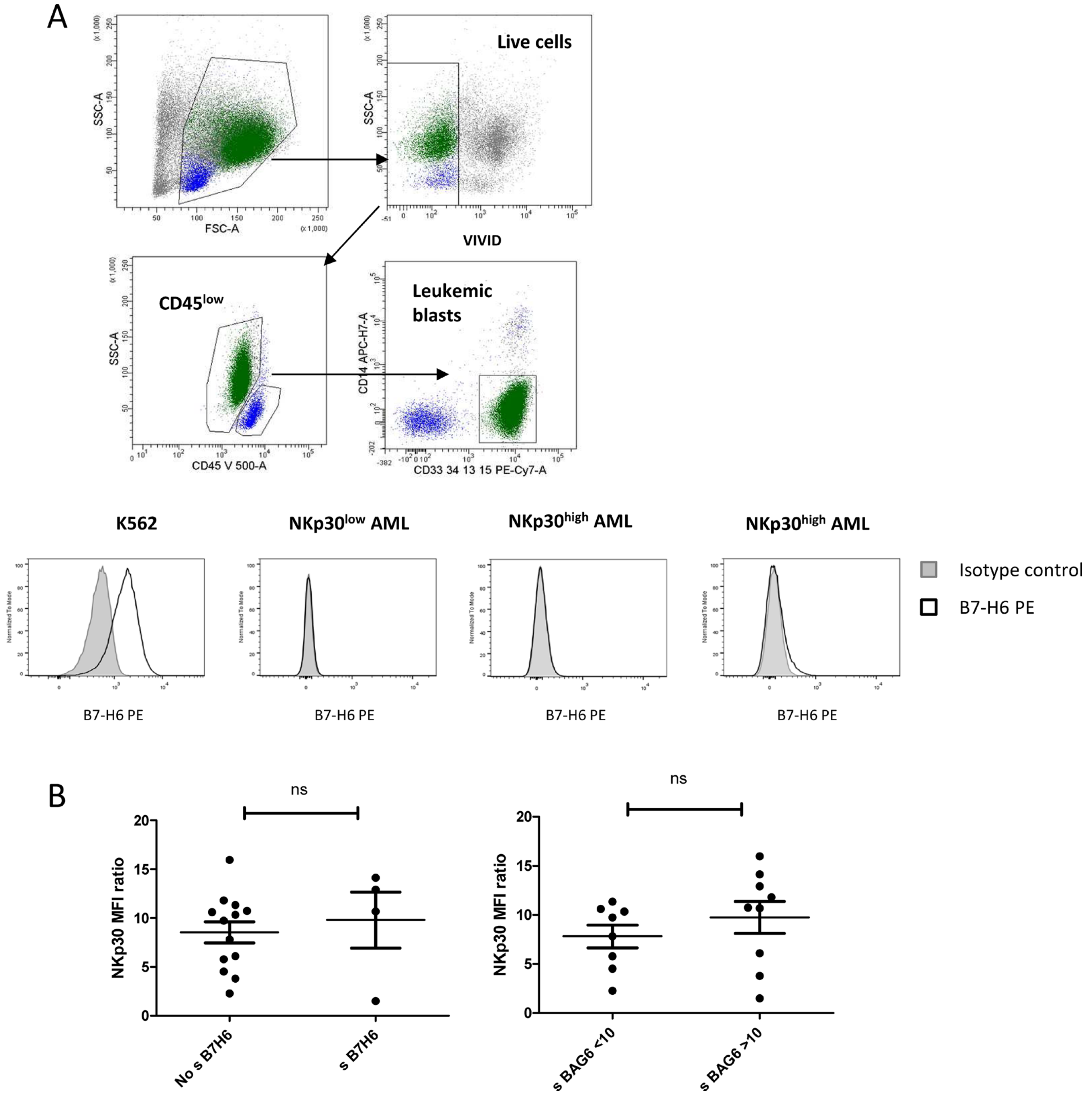

Supplementary Figure 3: NKp30 ligands. (**A**) NKp30 expression according to B7-H6 expresion on leukemic blasts: B7-H6 expression was detected on CD45low CD13+ and or CD33+ and or CD34+ cells by flow cytometry ($N = 39$ patients, GOELAMS cohort). NKp30 expression is represented in patients with no detectable B7-H6 expression, and in patients with at least 0.1% B7-H6+ leukemic blasts. K562 cell line was used a positive control. (**B**) NKp30 expression according to plasmatic NKp30 soluble ligands: soluble B7-H6 (sB7-H6) and soluble BAG6 (sBAG6) was detected in the plasma from 17 AML patients at diagnosis by ELISA. NKp30 expression was analyzed by flow cytometry on NK cells in patients with and without detectable soluble ligands. Statistical analyses were performed using a Mann Whitney test. $P < 0.05$ was considered significant. ns: non significant.
